# Effect of strip type denture adhesives on *Candida species* colonization in complete denture wearing patients

**DOI:** 10.6026/9732063002001613

**Published:** 2024-11-30

**Authors:** Sameer Chauhan, Paranjay Prajapati, Pritha Negi

**Affiliations:** 1 Department of Prosthodontics and Crown & Bridge, K. M. Shah Dental College and Hospital, Sumandeep Vidyapeeth, Piparia, Waghodia, Vadodara - 391760, Gujarat, India

**Keywords:** *Candida species*, complete denture, denture adhesive, denture retention

## Abstract

The present study was planned to evaluate the influence of strip of denture adhesive on oral microflora, mainly *Candida species* in the participants who were instructed to use Super Polygrip Comfort Seal Strips denture adhesive for 14 days of trial period. 24 completely edentulous patients treated with complete denture were randomly divided into following - Control and Test group (12 each group). Test group was prescribed a strip denture adhesive and instructed to use it for 14 days. Samples of saliva were collected from all participants (both control and test groups) on 0 day, 7th and 14th day. Collected samples were diluted with standard protocol and coated on culture media of Sabouraud dextrose agar and incubated for 48 hours at 37°C. The CFU/ml was analyzed from both groups. Collected data then analyzed statistically with Mann - Whitney U - Test where α kept at ≤ 5%. No significant statistical difference was found between the control and the test group after the testing duration of 14 days. Thus, results suggested that strip denture adhesive did not have any influence on the colonization of *Candida species* during 14 days of test period and it is safe for the use in complete denture wearing patients.

## Background:

Adequate retention and stability are primary requirements for the acceptance by the patients wearing complete denture. The clinical procedures should be performed in such a manner that the fabricated dentures should be providing maximum retention, peripheral seal and intimate contact with the oral tissues [1]. Though, in certain conditions, denture adhesives materials are prescribed to enhance denture function by enhancing basic requirements of prostheses like retention and stability [2, 3-4]. These materials are used to promote adhesion between mucosa and intaglio surface of denture. Nevertheless, previous studies mentioned that acrylic based dentures act as carrier for micro-oranisms in the oral cavity [5, 6-7]. Various surface properties of acrylic resin (poly methyl methacrylate-PMMA) such as surface topography, micro-roughness, surface free energy, hydrophobic nature and acid based component of the material have been contributing to the adhesion of microorganisms [8]. Possibilities of microbial adhesion to denture surface increases when denture adhesives are used due to its mechanism of action. These materials are usually composed of a synthetic polysaccharide with coloring agents other stabilizers. A denture adhesive generally works by absorbing water from saliva which led to increase viscosity and volume of the material. Thus such highly viscous volume of denture adhesive accommodates the space between the intaglio surface of the denture and mucosa and ultimately enhances its retention [9]. However, this viscous nature of denture adhesives contributes to the suitable environment for the microbial adherence.

Presently no data available about the number of users of denture adhesive materials in India but in United States of America, around 22 of complete denture users use denture adhesives on regular basis [10]. 75% dentists also recommend their completely edentulous patients to use denture adhesives [11]. Basic mechanism of the denture adhesives is to enhance retention and stability of the denture. It also enhances the masticatory ability and also prevents food lodgment between denture surface and mucosa. Though, denture adhesives are widely used materials by denture users, however it also encourages the patients to use poorly fitting dentures for the extended period of time and thus it causes alveolar bone resorption. It also interferes with the mastication and acts as an irritant for the oral mucosa [12- 13]. Furthermore, their effect on oral micro-flora, cytotoxic ability and prolonged use could further aggravate bone resorption and also causes hyperplasia in such patients [14, 15-16]. 20-50 % of normal healthy individuals evidently have *Candida species* in their oral cavity which goes up to 60 to 100% when complete denture patients are considered. Among all *Candida species*, the most commonly found species are Candida albicans and contribute for 70% colonization in the oral cavity [17-18]. Higher salivary count of Candida albicans is considered to be an aetiology and pre-disposing factor for denture stomatitis [19, 20, 21, 22, 23-24]. Strip type of denture adhesive has not been evaluated previously for its effect on colonization of *Candida species*. With this background, the present randomized controlled clinical study was planned to assess the outcome of strip type of denture adhesive on the colonization of *Candida species* in completely edentulous patients using complete dentures, after 14 days of customary use.

## Materials and Methods:

## Study design and inclusion of participants:

A Randomized Controlled Clinical study was planned to assess the effectiveness of strip type of denture adhesive on colonization of *Candida species* in complete denture wearing patients. Approval to conduct randomized controlled clinical study was obtained from the Institutional Ethics Committee (IEC) under protocol approval number SVIEC/ON/DENT/SRP/16574 of Sumandeep Vidyapeeth deemed to be University, Vadodara, India. The study was conducted between June 2021 to September 2022 in the Department of Prosthodontics and Crown & Bridge, K. M. Shah Dental College and Hospital, Vadodara, India. Based upon the values obtained from the previous study by Oliveria *et al.* which assessed the effectiveness of tape denture adhesive on the colonization of *Candida species* in completely edentulous denture wearing patients, a sample size of total 24 participants, 12 each group (control group and test group) was decided for the study with 1 Standard Deviation (SD), 95% confidence interval, 80% power and 20% of possible dropout [25]. A total 24 completely healthy individuals, age ranging from 45 to 70 years were included in the study with the following inclusion and exclusion criteria: Individuals who were not on any antibiotic medicine, no sign of Oral Candidiasis and who cleared the test for *Candida species* in saliva were included for the research. Patients aged below 45 years, with systemic disorders and patients previously used denture adhesives with complete dentures were excluded from the study. Participant's information sheet was provided to all participating patients which has objectives, risk and benefits to which they would be exposed during the course of study and informed consent was taken before the enrollment in the study. Both genders were considered during the study, but the gender was not considered as a selection criteria for the study.

## Protocol for microbiological evaluation:

Samples of 5 ml of non-stimulated saliva were collected from all the participants and stored into the sterile test tubes with appropriate identification. Samples of saliva were collected from all the participants from the control and test groups on 0 day, after 7 days and 14 days. Samples were collected and stored into closed containers until they were processed further. The collection and evaluation of samples were not extended beyond 14 days as the included participants in the control group would develop candidiasis after the increase of colony count. Moreover, in the previous studies, a standard duration of 14 days has been used as a standard time to evaluate *Candida species* [15, 16, 17, 18, 19, 20-21]. The samples of saliva were homogenized in an agitator to isolate the Candida colonies. The samples of saliva were generated with 10-1 dilution by adding 1 ml of salivary sample into 9 ml of sterile physiologic solution. The samples were further diluted up to concentration of 10-6 which is a satisfactory dilution to measure Candida colonies effectively. From this prepared dilution samples, 0.1 ml was spread on the surface of sterile Petri dishes having Sabouraud dextrose agar (SDA) and incubated in the closed oven with temperature control for 48 hours at 37°C temperature. After incubation period of 48 hours, the developed colonies were identified on SDA plates by their microscopic and macroscopic features. Typical colonies were identified by their sphere-shape, white color and dull finish, with a ceramic appearance and up to 8 mm diameter in size. The mean number of colonies from two petri dishes was then multiplied by the dilution factor and aliquots used for the preparation of samples to count Colony forming Units (CFUs) per ml.

## Study protocol:

**Figure 1:**
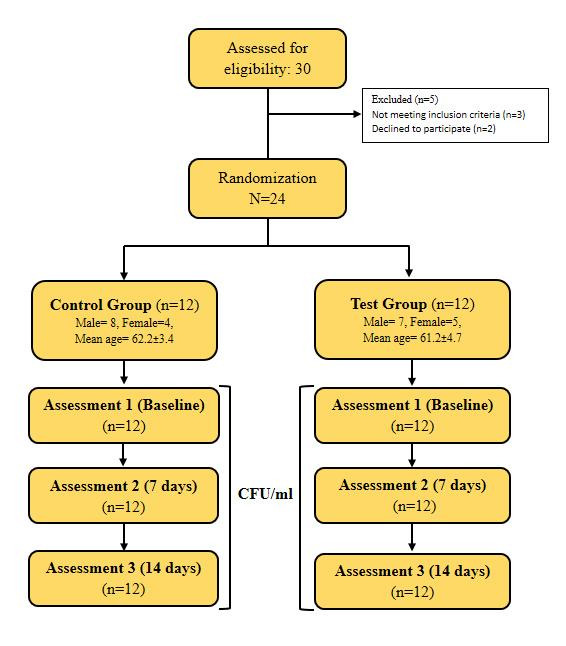
Application of strip type denture adhesive on the intaglio surface of maxillary and mandibular dentures

30 completely edentulous patients seeking complete denture treatment were screened for inclusion in the study. 6 patients were excluded from the study as they did not meet the inclusion criteria (3 patients) or refused to participate (2 patients). The 24 participants were distributed randomly into 2 study groups - Test and Control groups with 12 participants in each group. The random sequence for participants was generated using computer generated random numbers by another researcher who was not involved in the process of treatment provided. All the participants were provided with properly adapted new set of complete denture which was adequately retentive and stable during function. Super Polygrip Comfort Seal Strips (Super Polygrip, Glaxo Smith Kline GSK, Pennsylvania, PA, USA) was provided to the participants in the test group. They were instructed to apply adhesive strips on the residual ridge and posterior palatal seal area of maxillary denture and on the residual ridge area of mandibular denture (Figure 1). Pre-recorded video demonstration regarding the proper technique of application was shown to all the participants of test group. Participants in the test groups were instructed to apply adhesive strips in the morning time after cleaning the denture and they were asked to wear denture throughout the day without removing adhesive strips from dentures. All participants were given instructions about cleaning of dentures using coconut soap and denture cleaning brush, under the running water and the technique was demonstrated with a standard video presentation. Participants were also provided printed leaflet with instructions and pictorial presentation of proper application technique of adhesive strips and denture cleaning protocol.

## Statistical analysis:

Data collection was performed by another researcher who was not involved in the intervention procedures. The CFU counts for each participant at different time intervals were entered in the Microsoft Excel® spreadsheet, Microsoft Office 365, Version 2205. The mean difference between groups was calculated using SPSS (Statistical Package for Social Sciences) Version 20.1 (IBM Corp. Chicago, USA) software for the inter-group comparison by the non-parametric Mann - Whitney U test at the significance level of ≤ 5%.

## Results:

**Figure 2:**
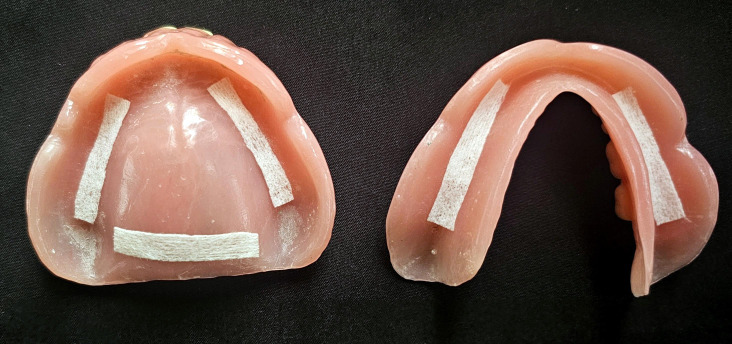
Flow diagram of participants (Adapted from the CONSORT statement)

The flow diagram of participants throughout the research is shown in Figure 2. It was adapted from the CONSORT guidelines for randomized control clinical study. Total 24 participants after assessing with inclusion and exclusion criteria were included in the study. Enrolled participants were comprised of 15 male (Control: 8, Test: 7) and 9 women (Control: 4, Test: 5). the general characteristics of participants are described in the Table 1 for both control and test group. Assessment of CFU/ml count for each participant was performed at 3 different time intervals: 0 days (baseline), 7 days and 14 days. The study protocol did not report any dropout during the test period of 14 days in both control and test groups. The mean and standard deviation (SD) of absolute CFU/ml of salivary samples are presented in the Table 2 for both the groups. No statistical significant difference was found in the mean values of CFU/ml for both control and experimental group. Table 3 presented the values of comparison of CFU/ml count at different time intervals. The difference in the CFU/ml count showed no statistically significant difference at the different time intervals. CFU/ml count in both the groups showed increase at the intermediate time interval of 7 days, however the change in CFU/ml count was not statistically significant (p = 0.73). Thus, result showed no statistical increase in CFU/ml and justified the use of strip type of denture adhesive for 14 days of experimental period.

## Discussion:

Denture related stomatitis is a common pathology among complete denture wearers which affects the palatal mucosa and also have erythematous characteristics. This condition is associated with the poor maintenance of denture which led to excessive colonization of Candida albicans on the intaglio surface of complete denture [14, 15 16, 17, 18, 19, 20, 21, 22-23]. Chemical configuration and topography of denture surfaces increase the chance of microbial adherence mainly on the intaglio surface of the denture [16- 18]. A property such as permeability and surface porosities of the denture base resin material is a primary reason for the adherence of the microorganisms to the denture base [21]. Use of denture adhesive materials with denture relining materials changes the topography of intaglio surface of dentures in contact with palatal mucosa. Nevertheless, the effect of strip type of denture adhesives on the number of CFUs of the *Candida species* has not been evaluated in complete denture wearing patients in India. The strip type of denture adhesives mainly consists of insoluble polypropylene fibers, cellulose and ethylene oxide or sodium alginate material. This composition turns into viscous material when it comes in contact with the water from the saliva [3]. Denture adhesive used in the present study has ethanol in the composition which has antifungal effect and this fact is supported by the manufacturer. This fact was not taken into consideration during the study as, both the control and test groups showed no statistically difference after experimental period of 14 days.

Result of the present study indicated that the use of Super Polygrip Comfort Seal Strips did not influence statistically the CFU count of *Candida species* after experimental period of 14 days, when it was compared with the same duration for control group where the use of any denture adhesive was prevented (Table 3). Regardless of few individual data from both the groups, no significant increase or decrease in CFU count was observed during the study and thus general trend could not be drawn at the end of experimental time of 14 days. Previous studies are available in the literature which experimented microbial growth in denture adhesives. Bartels *et al.* and Staforred *et al.* in their in vivo research reported no inhibitory action of certain powder type of denture adhesive on *Candida species* when tested on salivary samples [4, 12]. Moreover, Makihira *et al.* reported that two different brands of denture adhesives were capable of inhibiting the growth of Candida albicans due to the low pH established by such materials [13]. In the similar line of the in vivo evaluation of Kim *et al.* the present study presented no effect on the growth of *Candida species* [14]. However, the study was conducted with powder and paste type of denture adhesive and not with strip form. Scher *et al.* conducted their study on the participants with the inflamed oral mucous membrane and denture stomatitis and concluded that reduction in Candida albicans colonies was associated with the use of denture adhesive [16]. This result is not in line with the present study, however, this could be justified by the different methodology and type of adhesives used in the study. Thus, strip type of denture adhesive is safe for the use in complete denture wearing patients as it does not have any effect on colonization of *Candida species*. Thus, it provides an alternative to powder and paste type of denture adhesives. In the present study, all the patients were instructed to maintain oral hygiene, appropriate use of denture adhesive and removal of the same from denture and in addition they were informed about their participation on the study and treatment provided. These factors may have had influence on the results derived from the present study. All the participants in the present study were provided with the new complete dentures with proper adaptation to mucosa, adequate retention and stability. However, major denture wearing population uses denture adhesives to enhance retention and stability of old or poorly fitting dentures. Though the test group in the present study did not show statistically significant difference in the number of CFU count when compared to control group at the end of experimental period of 14 days, but the results were less homogenous in nature. This finding might suggest that some of the individuals may react differently while using this product or duration of the study was relatively short to stabilize the number of CFU count. Future randomized control trials should be planned with a longer experimental duration as these products are used for longer duration by the patients with complete dentures. Moreover, different commercially available denture adhesive products should be compared to check the effect of the adhesive use on oral microflora.

## Conclusion:

Within the limitations of the present study, the use of strip type of denture adhesive did not have any influence on the CFU count of *Candida species* when used in the complete denture wearers for the test period of 14 days. Thus, strip type of denture adhesive is safe for the use in complete denture wearing patients and it provides better alternative to powder and paste type of denture adhesives.

## Figures and Tables

**Table 1:** General characteristics of included participants according to the group allotted

**Parameters**	**Control Group (n = 12)**	**Test Group (n = 12)**
**Gender distribution**		
Male	8	7
Female	4	5
**Mean age**		
Mean age in years	62.2±3.4	61.2±4.7
**Habit of Smoking**		
Absent	9	11
Present	3	1

**Table 2:** Means and standard deviations of colony forming units per milliliter (CFU/ml) for both control and test group

	**Control Group (n=12)**		**Test Group (n=12)**		
Time intervals	Mean	SD*	Mean	SD*	p value**
Baseline, 0 day	139.6	18.9	137.8	15.93	0.83
Intermediate, 7 days	149.2	23.89	143.6	18.01	0.67
Final, 14 days	135.8	19.37	136.4	25.56	0.92
*SD: Standard Deviation
**p value derived from Mann-Whitney U-test.

**Table 3:** Comparison of CFU/ml count at different time intervals

**Mean difference**	**Control Group**	**Test Group**	p-value*
Intermediate to baseline	9.6	5.8	0.42
Final to intermediate	-13.4	-7.2	0.25
Final to baseline	-3.8	-1.4	0.79
*Value of p which is ≤ 0.05 derived from Mann-Whitney U-test.
CFU: Colony forming Unit.
